# The diagnosis and treatment of traumatic retroperitoneal hematoma

**DOI:** 10.12669/pjms.292.3168

**Published:** 2013-04

**Authors:** Fengbiao Wang, Fang Wang

**Affiliations:** 1 Fengbiao Wang, MD.Department of General Surgery, Tianjin 4th Centre Hospital, Tianjin, 300140, China.; 2 Fang Wang, MD. Department of Anesthesia, Tianjin 4th Centre Hospital, Tianjin, 300140, China.

**Keywords:** Traumatic retroperitoneal hematoma, Diagnosis, Treatment, Exploratory laparotomy

## Abstract

***Objective:*** To analyze our experiences in patients with traumatic retroperitoneal hematoma, and highlight the problems in diagnosis and treatment to facilitate the surgeons to make decision.

***Methodology:*** One hundred and eight patients of traumatic retroperitoneal hematoma treated in our institution from May 2008 to Jun 2012 were reviewed retrospectively. The data including patient’s age, type of injury, hospital stays, type of treatment, injured organs and mortality rate were collected.

***Results:*** In 108 patients, seventy-seven patients were male and 31 were female with a mean age of 36.5 years; eighty-seven patients sustained blunt trauma and 21 penetrating injury. Centro-medial hematoma was found in 31 patients, lateral hematoma in 36 patients, pelvic hematoma in 35 and pelvic-central hematoma in six patients. Eighty-three patients were treated surgically and 25 patients were treated conservatively. Six patients died and the mortality rate is 6.5%. Wound infection occurred in five patients, deep vein thrombosis in one patient, gastric fistula in two patients and duodenal fistula in one patient.

***Conclusion:*** Traumatic retroperitoneal hematoma is life-threatening condition, early diagnosis and correct treatment is of upmost importance. Mandatory exploration should be performed in cases of retroperitoneal hematomas resulting from penetrating injury, but the selection of treatment mode in blunt injury depends on the anatomical position of hematoma, visceral injury and the hemodynamic status of the patients.

## INTRODUCTION

Traumatic retroperitoneal hematoma is the common complication of abdominal or pelvic injuries. Retro peritoneum contains a number of visceral and vascular structures in the gastrointestinal, genitourinary, vascular, musculoskeletal and nervous systems.^1^ It may be potentially responsible for the occurrence of traumatic retroperitoneal hematoma, and makes the diagnosis and treatment of the fatal lesion complicated. A mortality rate of traumatic retroperitoneal hematoma is reported as high as 18-60% in English literatures.^2^ The early diagnosis and correct treatment are critical to decrease the mortality of the life-threatening lesion. In the recent years, despite the advances in surgical techniques, the diagnosis and treatment of traumatic retroperitoneal hematoma still remain challenging^3^^,^^4^ and many uncertain points available.

Therefore, we reviewed retrospectively 108 patients of traumatic retroperitoneal hematoma treated in our institution between May 2008 and Jun 2012. The purpose of this study was to (1) analyze our experiences in patients with traumatic retroperitoneal hematoma and (2) highlight the problems in the diagnosis and treatment of the fatal lesion.

## METHODOLOGY

One hundred and eight patients of traumatic retroperitoneal hematoma treated in our institution from May 2008 to Jun 2012 were reviewed retrospectively. The data including patient’s age, type of injury, hospital stay, type of treatment, injured organs and mortality rate were collected.

Our study population consisted of patients in which the traumatic retroperitoneal hematoma was confirmed with a final diagnosis, and the cases of retroperitoneal hematoma resulting from other reasons were excluded from the study. Diagnosis was made by using ultrasonography, computed tomography (CT) or confirmed in exploratory laparotomy. According to the classification from Selivanov et al., the retroperitoneal hematomas were classified under three zones, i.e., centro-medial (zone I), lateral (zone II) and pelvic hematoma (zone III).^5^

The current study was approved by the Institutional Review Board and 108 patients were identified with International Classification of Diseases, 9th revision codes consistent with retroperitoneal hematoma.

## RESULTS

One hundred and eight patients included in the current study consisted of 77 men and 31 women with a mean age of 36.5 years (range from 15 to 54 years). The cause of trauma was automobile accident in 48 cases, fall in 19 cases, strikes injuries in 28 and crush injury in 13 cases. Eighteen patients were found single site injury, ninety patients multiple injuries; Eighty-seven patients sustained blunt trauma, twenty-one sustained penetrating injury; Centro-medial hematoma was found in 31 patients, lateral hematoma in 36 patients, pelvic hematoma in 35 and pelvic-central hematoma in six patients.

Ultrasonography was performed in 92 patients and 48 patients were diagnosed with retro peritoneal hematoma; Computed tomography (CT) was performed in 75 patients and diagnosis of retro peritoneal hematoma was confirmed in 64 patients. Diagnosis of twenty-two patients was confirmed on exploratory laparotomy.

In 108 cases, eighty-three patients were treated surgically and 25 cases conservatively. The type of operation in 83 patients are presented in Table-I. The retroperitoneal space was explored in 31 cases. In 25 patients with nonsurgical treatment, selective angiographic embolization of renal artery was performed in two cases, and bilateral angiographic embolization of internal iliac artery were performed in one patient, all of them survived.

**Table-I:** Operation performed in 83 patients

***The dead(n=6)***		***The survivors (n=77)***	
*Operation performed for the management of retroperitoneal hematoma:*			
inferior vena cava repair	1	Packing	14
abdominal aorta repair	3	repair of abdominal aorta	3
packing + ligation of internal iliac artery	1	Ligation or repair of internal iliac artery	4
		repair of common iliac vessels	3
*Associated operations performed:*			
splenectomy	1	Splenectomy	4
colostomy	1	Gastrorrhaphy	3
pelvic fixation using external fixator	1	Drainage or repair of pancreas	4
pelvic internal fixation	1	repair or partial resection of liver	6
		repair of duodenum	4
		repair of mesenterium	1
		repair or nephrectomy of kidney	3
		colostomy of left colon	4
		repair of right colon	3
		repair of ureter	4
		pelvic fixation	25
		repair or colostomy of rectum	3
		repair of urinary bladder	4
		Internal fixation for limb fracture	4
		Evacuation of subdural hematoma	2
		Thoracotomy	1

The mean hospital stays was 9.2 days (range from six to 16 days) for the conservative treatment and 13.6 days (range from 10 to 24 days) for surgical treatment. The mean follow-up was eight months, ranging from two months to 19 months.

In 108 cases, one hundred and two patients survived. Six patients died in surgical exploration, among which one patient had been suffering from inferior vena cava injury, two had been suffering from pelvic hematoma and three from abdominal aorta injury. The mortality rate was 6.5%.

In addition, infection, deep vein thrombosis, gastric and duodenum fistula were also noted. Wound infection occurred in five patients, deep vein thrombosis in one patient, gastric fistula in two patients and duodenum fistula in one patient. Wound infection was treated by daily dressings, gastric or duodenum fistula was treated by drainage, and the case of deep vein thrombosis was performed embolectomy.

## DISCUSSION

Traumatic retroperitoneal hematoma is a common, life-threatening complication of abdominal or pelvic injuries, early diagnosis and urgent surgical intervention are of utmost importance. In the current study, we performed a review of 108 cases treated in our institution to help surgeons determine the strategy of diagnosis and treatment for the fatal lesion.

In terms of the diagnosis, the signs and symptoms of traumatic retroperitoneal hematoma include abdominal pain, abdominal distension, abdominal mass, severe back and lower quadrant pain and femoral neuropathy, all of which is nonspecific, leading to the difficulties in diagnosing traumatic retroperitoneal hematoma according to clinical features.^3^ CT and ultrasonography play an important role in the assessment of retroperitoneal organs,^6^^,^^7^ facilitating greatly the diagnosis of traumatic retroperitoneal hematoma, helping surgeons make treatment decision. While, ultrasonography can’t accurately detect the extent or exact site of organ injuries, in addition to that, its sensitivity for direct demonstration of abdominal injury is relatively low.^8^ In the current study, out of ninety-two patients who underwent ultrasonography examination only 48 patients were diagnosed with retroperitoneal hematoma. Therefore, hemodynamically stable patients with a negative diagnosis from ultrasonography and a high clinical suspicion of abdominal injury should undergo routine CT scanning.

Although there are many advantages in CT, some factors such as the size and position of hematoma, experience of radiologists and resolution of CT may affect its diagnostic accuracy. In the current study, CT examination was performed in 75 cases and 64 were diagnosed with traumatic retroperitoneal hematoma. Subsequently, we suggest the exploratory laparotomy is the primary and safe method to diagnose the fatal lesion, especially in patient with hemodynamic instability.

There are two treatment approaches for traumatic retroperitoneal hematoma, operative and conservative.^9^ Retroperitoneal hematoma results from the ruptured solid organs, retroperitoneal blood vessels or associated with injuries of intra peritoneal organs. In our opinion, once the injury of organs was confirmed, exploratory laparotomy should be performed without delay. The sources of hemorrhage and natural history of the hematoma differ considerably depending on the etiology.^1^ In cases of penetrating injury, most of traumatic retroperitoneal hematomas may be accompanied with abdominal visceral injury, and exploratory laparotomy should be performed immediately. In case of blunt injury, when the organ injuries can’t be diagnosed definitely, whether the exploratory laparotomy should be performed or not, depend on the clinical status of hematoma. Presence of an expanding hematoma, pulsatile mass, and uncontained abdominal mass indicate need for surgical exploration.^9^

Moreover, retroperitoneal hematoma in different anatomical position has different clinical features and treatment strategy. The retroperitoneal hematoma in centro-medial zone is usually the consequence of the injury of duodenum, pancreas or great vessels. The presence of progressive sign and symptoms, increased amylase in blood and urine, free gas within the abdominal cavity and effusion around duodenum or pancreas indicate the injury of duodenum or pancreas, exploratory laparotomy need to be performed. In the current study, pancreas injury was confirmed in four cases and the pancreas repair and drainage were done urgently, all the patients recovered and were discharged. On the other hand, we suggest the stable hematoma without injury of organs in the centro-medial zone be managed using conservative approach, but the patients should be monitored closely.

Compared with the retroperitoneal hematoma in centro-medial zone, the necessity of urgent operation is not so high in patients with retroperitoneal hematoma in lateral zone. In the zone, we found most of the retroperitoneal hematomas were accompanied with injury of kidney, followed by injury of colon. The perirenal retroperitoneal hematoma resulted from blunt trauma can be treated conservatively and most patients survived.^1^ However, should the hematoma rapidly expand, become pulsatile, or rupture, it is usually opened via emergency operation. Twenty cases of perirenal retroperitoneal hematoma in the current study were treated non-surgically and three cases were treated surgically. Out of the three cases, renal resection was performed in two and repair was performed in one case for the serious damage, all cases survived. As regards the hematoma beside colon, we suggest exploratory laparotomy be performed to avoid the missed diagnosis of colon injury.

In the current study, the most common type of retroperitoneal hematoma was located in pelvic zone, the primary cause of which is pelvic fracture. The bleeding may cease after appropriate resuscitation and pelvic stabilization, while persistent haemodynamic instability may be found in some patients. The effect of angiographic embolization and packing on hemodynamically unstable multiple trauma patients with pelvic injury has been highlighted by some authors. In the current study, twenty-seven cases of pelvic fracture had fixation using internal fixation or external fixator. We found the haemodynamics of patients became stable after pelvic fixation except four patients, in which angiographic embolization of bilateral internal iliac artery was performed in two patient, ligation of internal iliac artery together with packing was performed in two patients. Two patients survived, but two cases died of hemorrhagic shock. Tolga et al suggest hematoma in the retroperitoneal space can be taken under control to an extent by applying pressure on the bleeding region, while exploratory laparotomy for hematoma may result in uncontrollable bleeding, even death of the patients. We support the above mentioned viewpoint and in our cases, most retroperitoneal hematomas in pelvic zone were not explored. However, when the retroperitoneal hematomas were accompanied with concomitant injury of rectum, bladder or other organs, surgical exploration is critical.

In conclusion, traumatic retroperitoneal hematoma is life-threatening lesion and early diagnosis and correct treatment are of utmost importance. We suggest that mandatory exploration should be performed in retroperitoneal hematomas resulted from penetrating injury, but the selection of treatment mode in blunt injury depend on the anatomical position of hematoma, visceral injury and the hemodynamic status of patients. 
